# Food insecurity and coping strategies and their association with anxiety and depression: a nationally representative South African survey

**DOI:** 10.1017/S1368980023000186

**Published:** 2023-04

**Authors:** Siphiwe N Dlamini, Ashleigh Craig, Asanda Mtintsilana, Witness Mapanga, Justin Du Toit, Lisa J Ware, Shane A Norris

**Affiliations:** 1 SAMRC/Wits Developmental Pathways for Health Research Unit, School of Clinical Medicine, Faculty of Health Sciences, University of the Witwatersrand, Johannesburg 1862, South Africa; 2 DSI-NRF Centre of Excellence in Human Development, School of Public Health, University of the Witwatersrand, Johannesburg, Gauteng, South Africa; 3 Global Health Research Institute, School of Health and Human Development, University of Southampton, Southampton, UK

**Keywords:** Food insecurity, Coping strategies, South African survey, Anxiety, Depression

## Abstract

**Objective:**

To investigate food insecurity and related coping strategies among South African households and their associations with anxiety and depression.

**Design:**

Cross-sectional study. Food insecurity and coping strategies were assessed using a modified Community Childhood Hunger Identification Project and the Coping Strategies Index questionnaires. The Generalized Anxiety Disorder-7 and Patient Health Questionnaire-9 were used to assess anxiety and depression risk. Ordered logistic regressions were used to test associations of food insecurity and related coping strategies with anxiety and depression.

**Setting:**

South Africa during COVID-19, October 2021.

**Participants:**

Nationally representative sample of 3402 adults, weighted to 39,640,674 South African households.

**Results:**

About 20·4 % of South African households were food insecure, with the most affected being from the lowest socio-economic groups. Shifting from ‘food secure’ to ‘at risk’ or from ‘at risk’ to ‘food insecure’ group was associated with 1·7 times greater odds of being in a higher category of anxiety or depression (*P* < 0·001). All coping strategies were used to some extent in South African households, with 46·0 % relying on less preferred and less expensive foods and 20·9 % sending a household member to beg for food. These coping strategies were mostly used by food-insecure households. Although the odds of moving to a higher category of anxiety and depression were observed among all coping strategies (all *P* < 0·001), begging for food was associated with the highest odds (OR = 2·3).

**Conclusions:**

Food insecurity remains a major health threat in South Africa. Public measures to address mental health should consider reductions in food insecurity as part of their strategy.

Food insecurity—the state in which households lack access to sufficient food due to limited money or other resources—is associated with negative health outcomes, including increased chronic disease risk, malnutrition and mortality rate^(1)^. While food insecurity remains a major global issue, middle-income countries such as South Africa are disproportionally affected^(2)^. For example, in 2017, over 21·0 % of South African households (*v*. 11·8 % in the USA, a high-income country) had inadequate food access, rendering food insecurity as one of the nation’s leading health and nutrition issues^(3,4)^. Due to recent economic disruptions associated with the COVID-19 pandemic, the high prevalence of food insecurity in South Africa may have worsened. Findings from the National Income Dynamics Study-Coronavirus Rapid Mobile (NIDS-CRAM) survey suggested that in March 2021 about 35·0 % of the South African population ran out of money to buy food^(5)^. The same survey reported an increased risk of screening for depressive symptoms from 21·0 % in 2017 to 29·0 % in 2021, with food-insecure households being the most affected^(6)^. With more recent developments between Russia and Ukraine, basic food prices have significantly increased in South Africa, and this may have further exacerbated food insecurity^(7)^.

There are a variety of strategies that households often use to cope with food insecurity. These include eating less expensive food, borrowing food or money, using credit, relying on relatives or friends, limiting portion sizes or the number of meals per day and even begging for food^(8)^. However, the impact of such coping strategies on mental health is less documented. Considering the vast inequalities in socio-economics in South Africa^(9)^, nationally representative studies are needed for improved estimates of the prevalence of food insecurity and its associated coping strategies.

Therefore, the aim of this study was two-fold: (i) to use a nationally representative survey in determining the prevalence of food insecurity and related coping strategies among South African households during low levels of restrictions (post wave 3 of the COVID-19 pandemic) and (ii) to assess the impact of food insecurity and related coping strategies on risk of anxiety and depression among South African adults.

## Methods

### Study design and setting

**Fig. 1:**
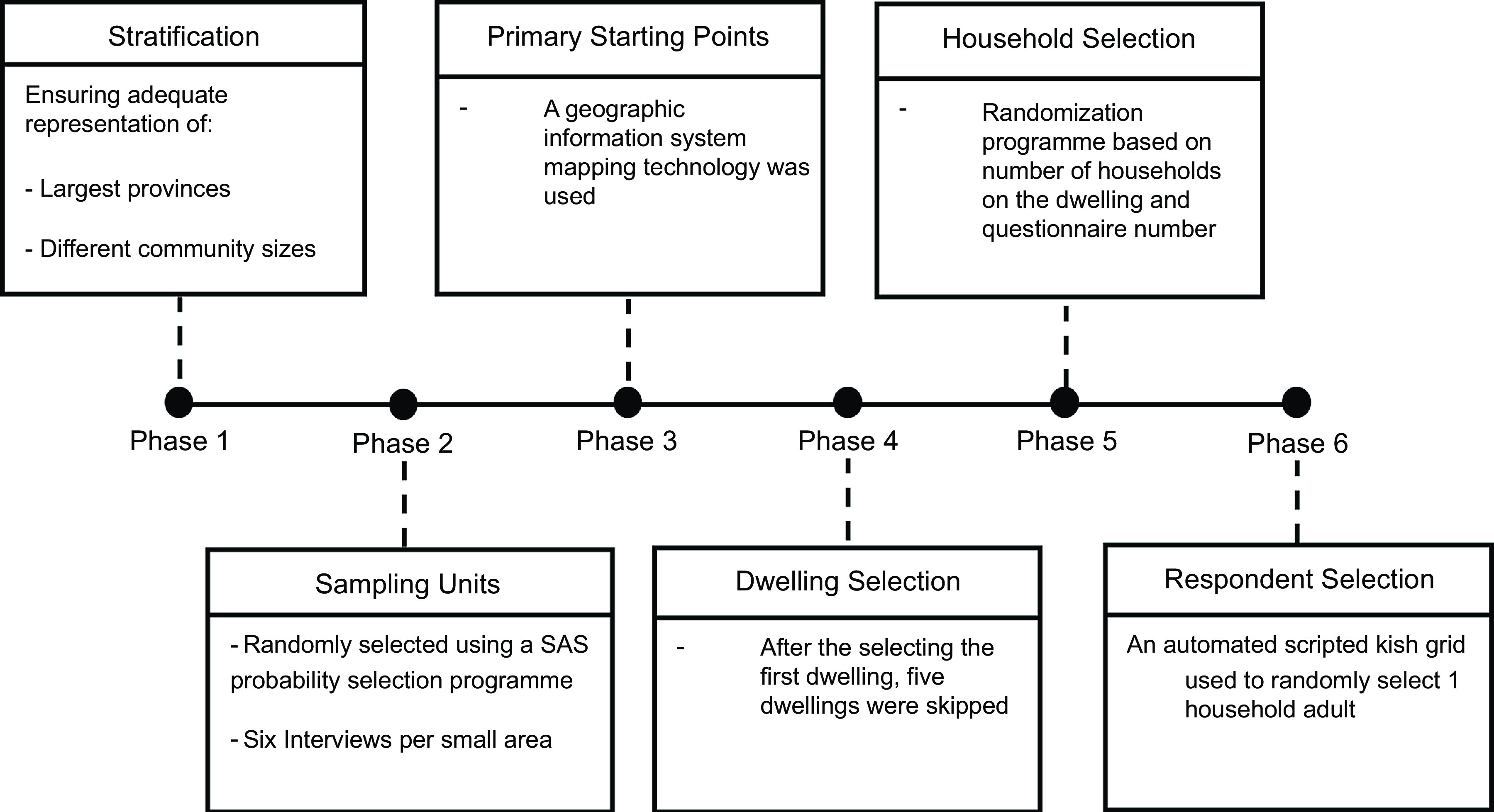
The six-phase stratified random probability sampling approach was used in the study. Phase 1 stratification was three-staged, to ensure adequate representation of the (i) largest provinces, (ii) different community sizes, (iii) and gender. Phase 2 involved the selection of sampling units, defined as small areas with merging smaller Enumerated Areas and with population sizes greater than 500. During this stratification phase, a SAS probability selection programme was used to randomly select the sampling units based on sampling proportionate to size. Six interviews were conducted per small area. Phase 3 involved using a geographic information system mapping technology to randomly select starting points, which were generally schools, churches, or prominent buildings from which the interviewer started their random walk. Phase 4 involved dwelling selection. From the identified starting point, interviewers went up the road based on the number indicated on the dwelling walls increasing. While keeping to the left side of the road, the interviewers turned left as long as the street formed part of the selected small area. Once the first dwelling was selected, five dwellings were skipped, and the interview was conducted at the sixth dwelling. Phase 5 involved household selection. A household was defined as a unit consisting of either one person living alone or a group of people–usually, but not always, members of one family who live together for at least four nights a week and whose food and other expenses are managed as one. The randomisation programme used to select the household was based on the total number of households in the dwelling and the questionnaire number. Phase 6 involved respondent selection. Once the household was identified; all household members, from the youngest to the oldest, were listed on a scripted kish grid. Excluding members younger than 18 years, the automated kish grid then selected the respondent in the household to be interviewed

A cross-sectional nationally representative survey comprising 3402 adults (52·5 % females, mean age 37·7 ± 12·3 years) was conducted in South Africa in October 2021. A six-phase stratified random probability sampling approach was used, with each phase summarised in Fig. 1. All field staff conducted face-to-face interviews using computer-assisted personal interviewing technology.

### Survey

The questionnaire included sections related to households (province, community type, household assets, food insecurity and coping strategies) and household respondents (age, gender, home language, ethnicity, employment status, level of education, personal income, mental health quality). To compute the household asset score, households were given a score of one for having each of the following 21 assets: (1) tap water in the house or on plot, (2) hot running water from a geyser, (3) built-in kitchen sink, (4) flush toilet in or outside the house, (5) deep freezer-free standing, (6) dishwashing machine, (7) refrigerator or combined fridge/freezer, (8) electric stove, (9) microwave oven, (10) tumble dryer, (11) vacuum cleaner or floor polisher, (12) washing machine, (13) computer-desktop/laptop, (14) DVD player or Blu Ray player, (15) home theatre system, (16) television (TV) set, (17) pay TV (Mnet/DSTV/StarSat) subscription, (18) air conditioner (excluding fans), (19) permanent swimming pool, (20) home security service and (21) home telephone (excluding cell phone).

An adapted Community Childhood Hunger Identification Project questionnaire was used for assessing food insecurity^(10,11)^. Questions assessing food coping strategies were from the Coping Strategies Index questionnaire^(12)^. The Patient Health Questionnaire-9 (PHQ-9)^(13)^ and Generalized anxiety disorder (GAD-7)^(14)^ were used for anxiety and depression screening.

### Definition of food insecurity, anxiety and depression groups

Although four questions from the Community Childhood Hunger Identification Project questionnaire were included in the study, only the following three were used to compute a food insecurity score (a score of 1 was given for each ‘Yes’ response)^(11)^:

1. ‘Does your Household ever run out of money to buy Food?’
2. ‘Do you ever cut the size of meals or skip meals because there is not enough money for food?’
3. ‘Do you or any of your children ever go to bed hungry because there is not enough money to buy food?’

The remaining question, ‘Do your children ever say they are hungry because there is not enough food in the house?’, was not used in the food insecurity score as it related to child hunger (This is reported separately). Household respondents who answered ‘No’ to all three questions (score = 0) were classified as ‘Food Secure’. Those who responded with a ‘Yes’ to only one of the three questions (score = 1) were classified as ‘At Risk’, while respondents who answered ‘Yes’ to two or three questions (score = 2 or 3) were classified as ‘Food Insecure’.

The GAD-7 total scores were used to classify the respondents into minimal (0 to 4), mild (5 to 9), moderate (10 to 14) and severe (15 to 21) anxiety groups^(14)^. Likewise, the PHQ-9 total scores were used to categorise the respondents into minimal (0 to 4), mild (5 to 9), moderate (10 to 14), moderately severe (15 to 19) and severe (20 to 27) depression groups^(13)^. These categories were subsequently used as outcomes in the statistical regression models.

### Statistical analyses

Data analyses were conducted in STATA 17·0. All descriptive data and regression models were weighted to represent the most recent census of a South African population (*n* 39 640 674; aged 18+ years) using a random interactive method^(15,16)^. The variables included in the weighting matrix were age, gender, province, ethnicity and home language.

All basic associations were tested using ordered logistic regression models and not adjusted for any confounder. For the associations of food insecurity, food insecurity group (food secure = 0, at risk = 1 and food insecure = 2) was the predictor variable while anxiety (minimal = 0, mild = 1, moderate = 2 and severe = 3) and depression groups (minimal = 0, mild = 1, moderate = 2, moderately severe = 3 and severe = 4) were the outcomes. Similarly, for the associations of coping strategies, the scores from each coping strategy were included as the predictors while anxiety and depression groups were the outcome variables. To estimate its independent effects, the coping strategy that most strongly predicted anxiety and depression was also tested in the regression model, while including all the other coping mechanisms as confounders.

## Results

### Prevalence of food insecurity among all South African households

**Fig. 2:**
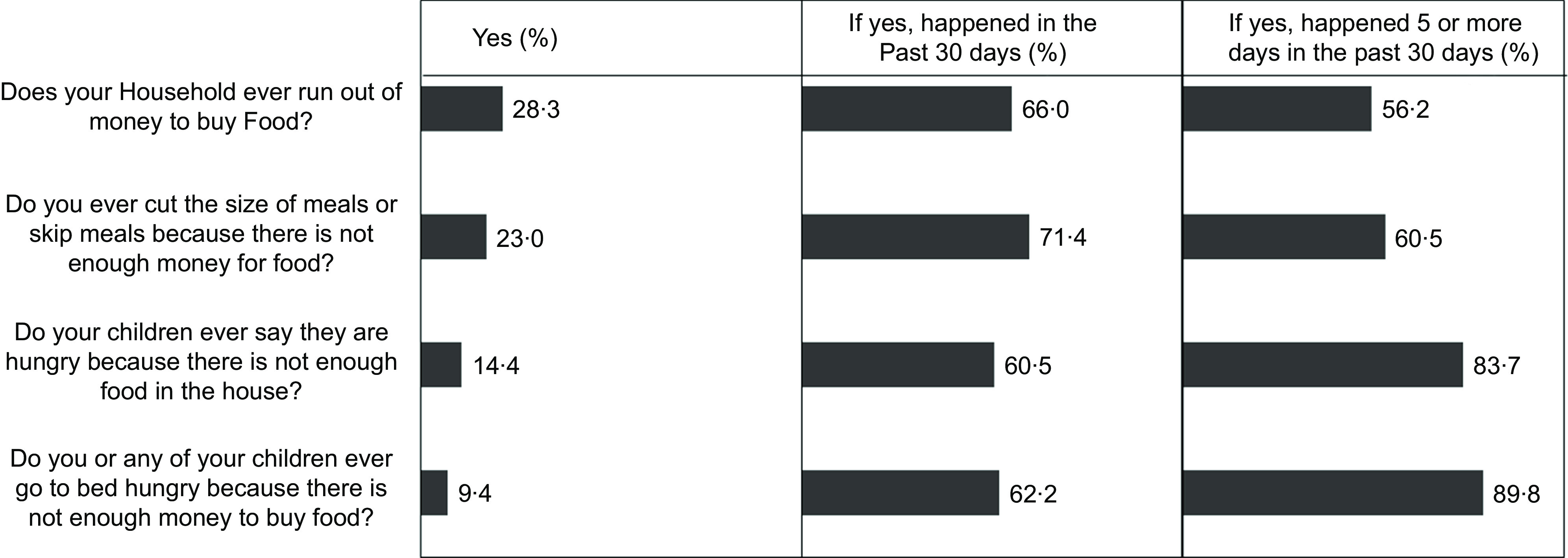
Responses to the food insecurity questions. Only respondents who answered yes to the previous question were asked the follow-up question (e.g. Happened in the past 30 d?)

**Fig. 3:**
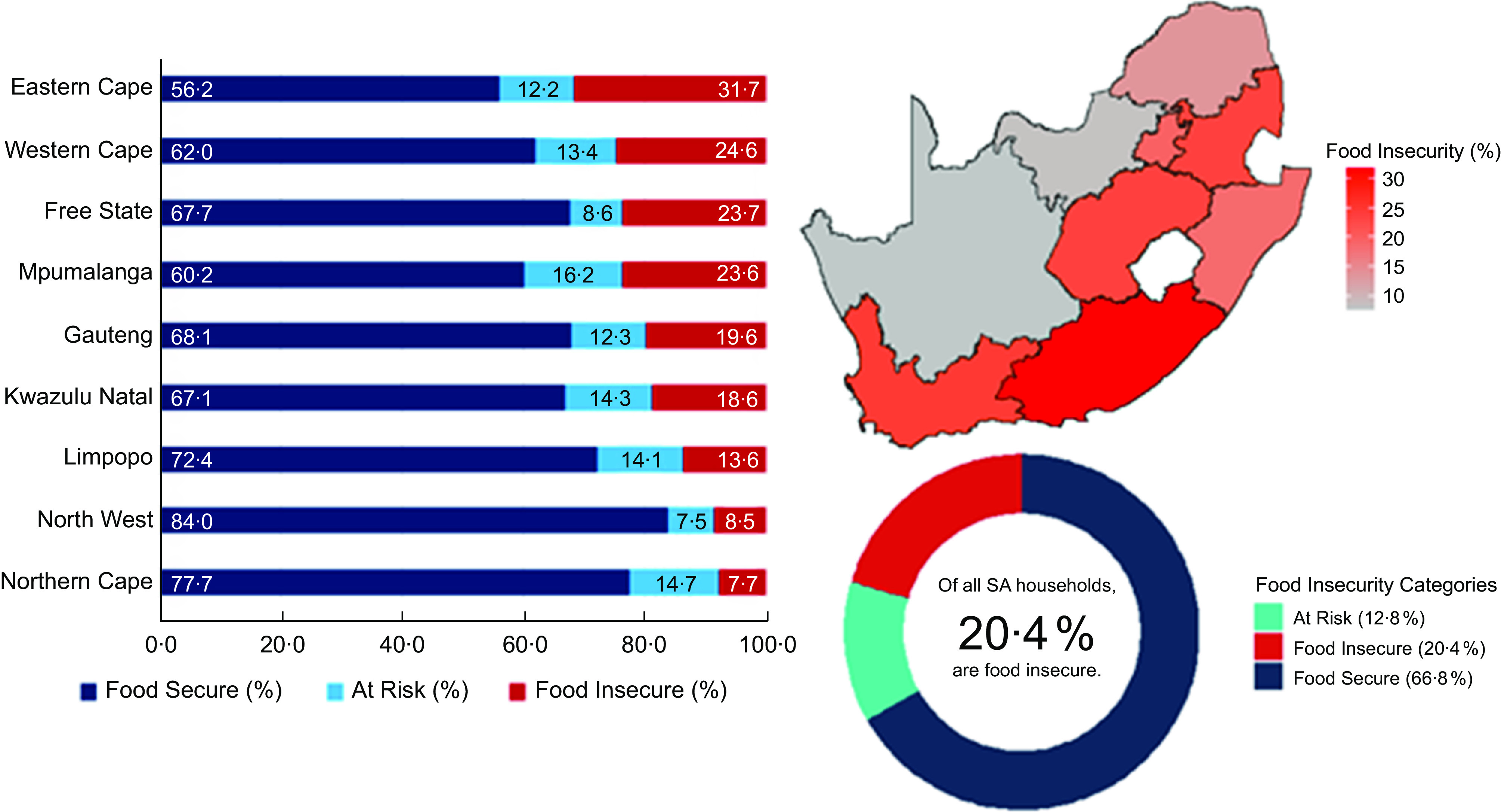
Prevalence of food insecurity among South African households

Responses to the four food insecurity questions are summarised in Fig. 2. A large proportion of all South African households experienced some form of food insecurity risk. For example, about 28·3 % of all households reported that they often run out of money to buy food. Notably, the majority of households (56·2 to 89·8 %) who were faced with food insecurity-related problems experienced them frequently, often five or more days in a month (Fig. 2). Figure 3 summarises the prevalence of food insecurity across all South African provinces. The province with the highest rate of food insecurity was the Eastern Cape (31·7 %), while the Northern Cape had the lowest food insecurity rate (7·7 %). Overall, 20·4 % (about 1 in 5) of all South African households were classified as food insecure.

### Food insecurity and socio-demographics

**Fig. 4:**
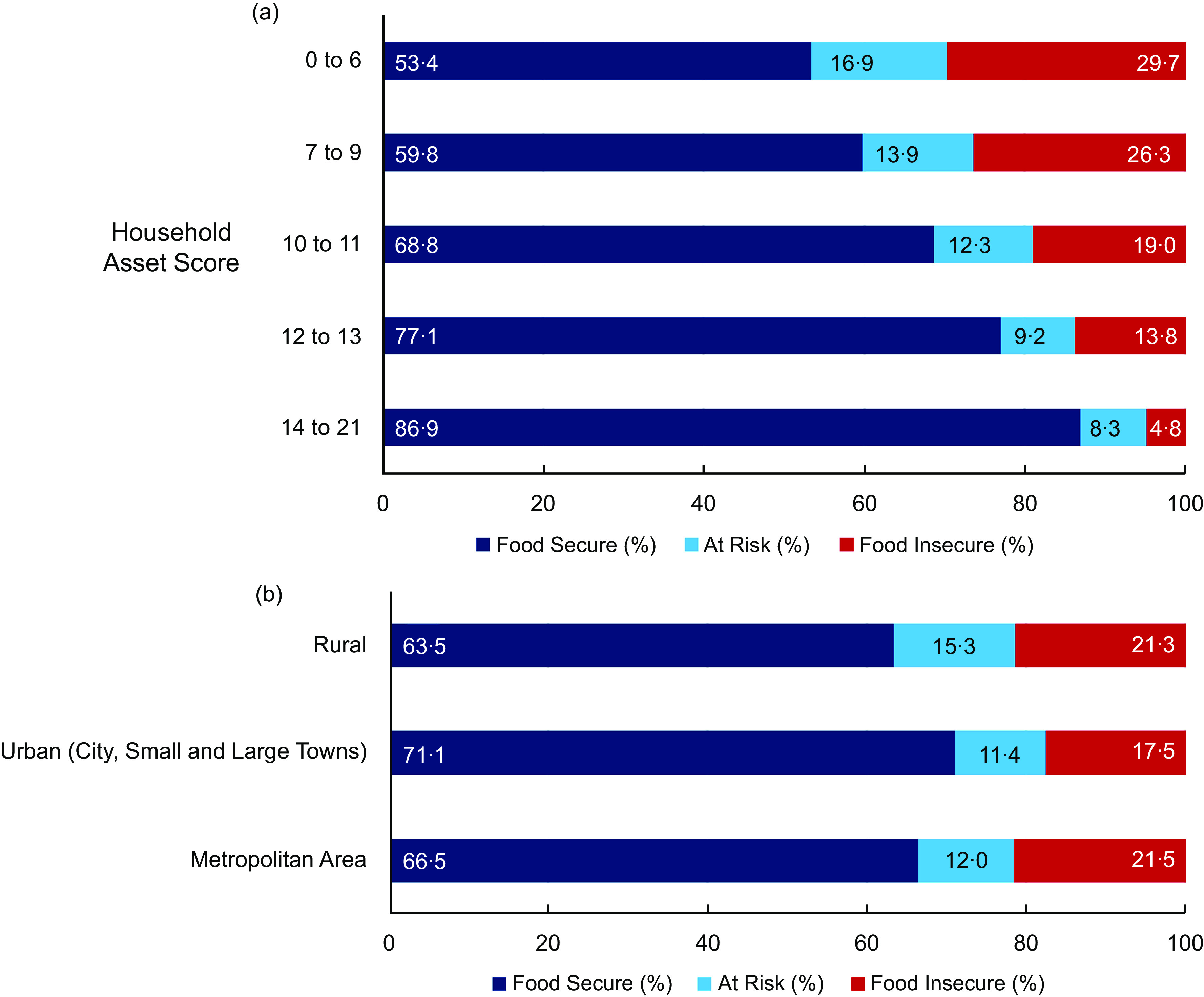
Food insecurity prevalence by household asset score (a) and community type (b)

**Fig. 5:**
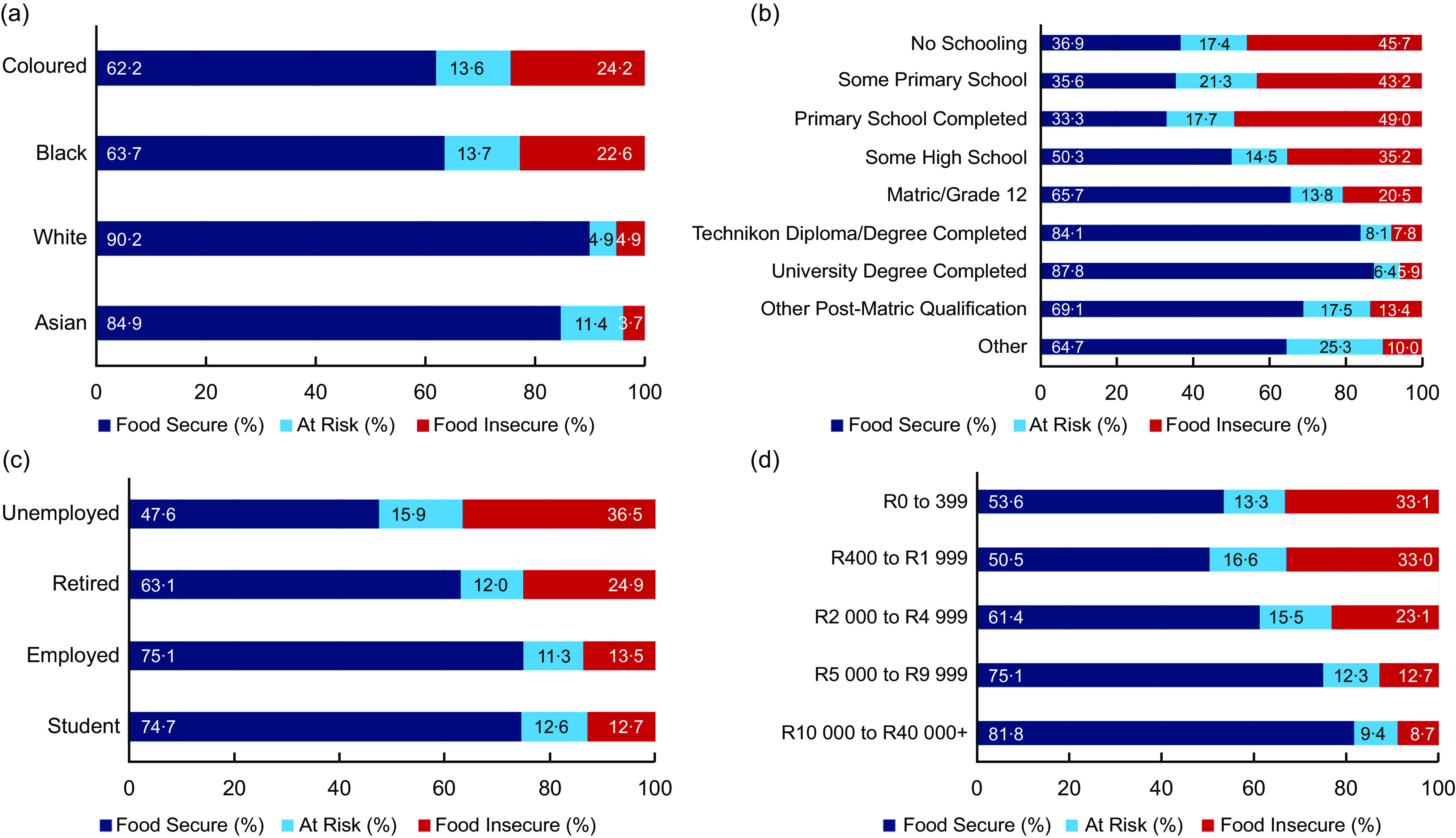
Food insecurity prevalence by respondent’s ethnicity (a), education level (b), employment status (c) and monthly income (d). Other: No formal education but has a short course certificate

Food insecurity is summarised by socio-economic factors in Figs 4 and 5. An increase in the household asset score was associated with a lower prevalence of food insecurity (Fig. 4(a)). Moreover, food insecurity was higher among households from rural (21·3 %) and metropolitan (21·5 %) areas when compared with households in urban (17·5 %) areas (Fig. 4(b)). Furthermore, the prevalence of food insecurity was much higher among Coloured and Black South Africans (24·2 % and 22·6 %, respectively) compared to their White and Asian counterparts (4·9 % and 3·7 %, respectively, Fig. 5(a)). There was a trend of lower food insecurity prevalence with increasing educational attainment, such that 45·7 % of respondents with no school were from food insecure households (Fig. 5(b)). Unemployed respondents were the most affected compared to the other employment status groups (e.g., 36·5 % for the unemployed *v*. 13·5 % for the employed, Fig. 5(c)). An increase in the respondent’s monthly income was also associated with a lower prevalence of food insecurity (Fig. 5(d)).

### Food insecurity coping strategies among South African households

**Fig. 6:**
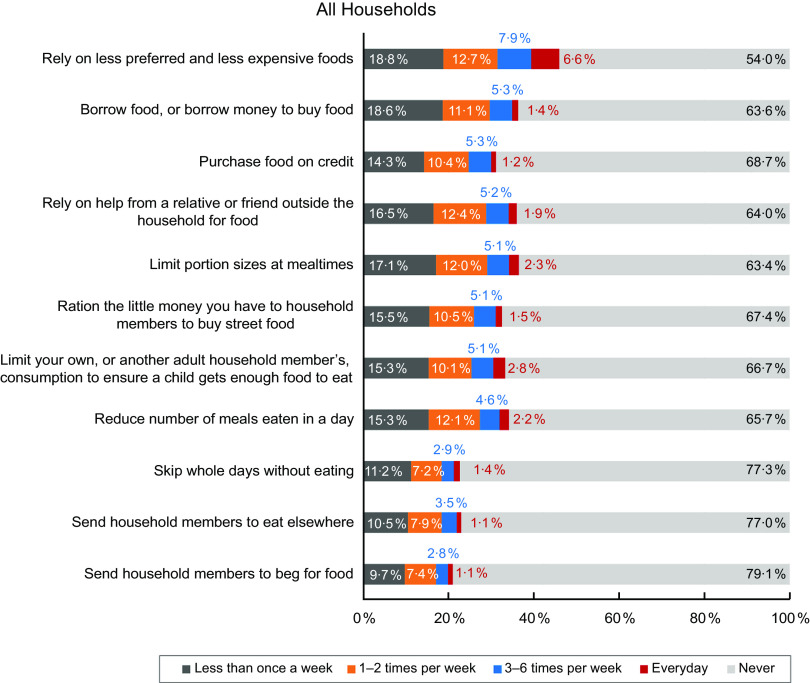
Coping strategies that prevailed among all households

Figure 6 summarises food insecurity coping strategies that were often used by South African households, with the frequency of use ranging from ‘never’ to ‘everyday’. While households used different coping strategies to deal with food insecurity-related issues, the most common coping strategy (used by 46·0 % of all households) was relying on less preferred and less expensive foods (Fig. 6). Conversely, sending a household member to beg for food was the least used strategy (20·9 % of all households, Fig. 6).

**Fig. 7:**
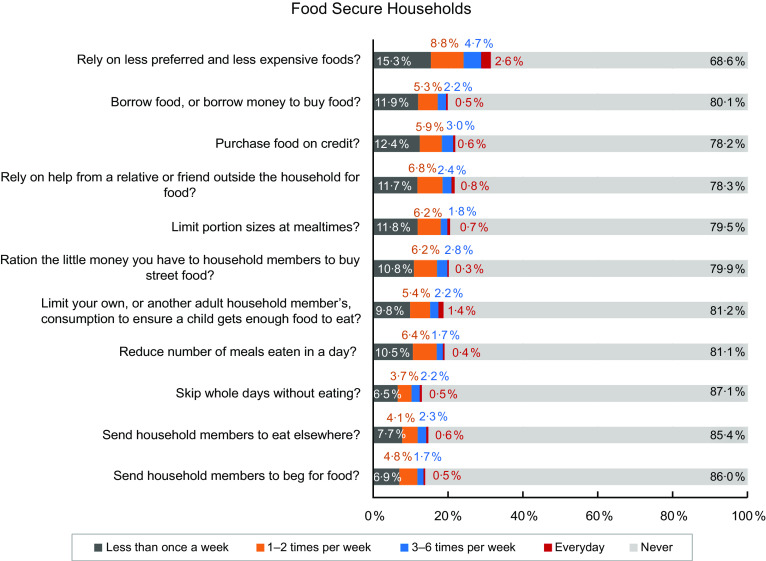
Coping strategies that prevailed among food-secure households only

**Fig. 8:**
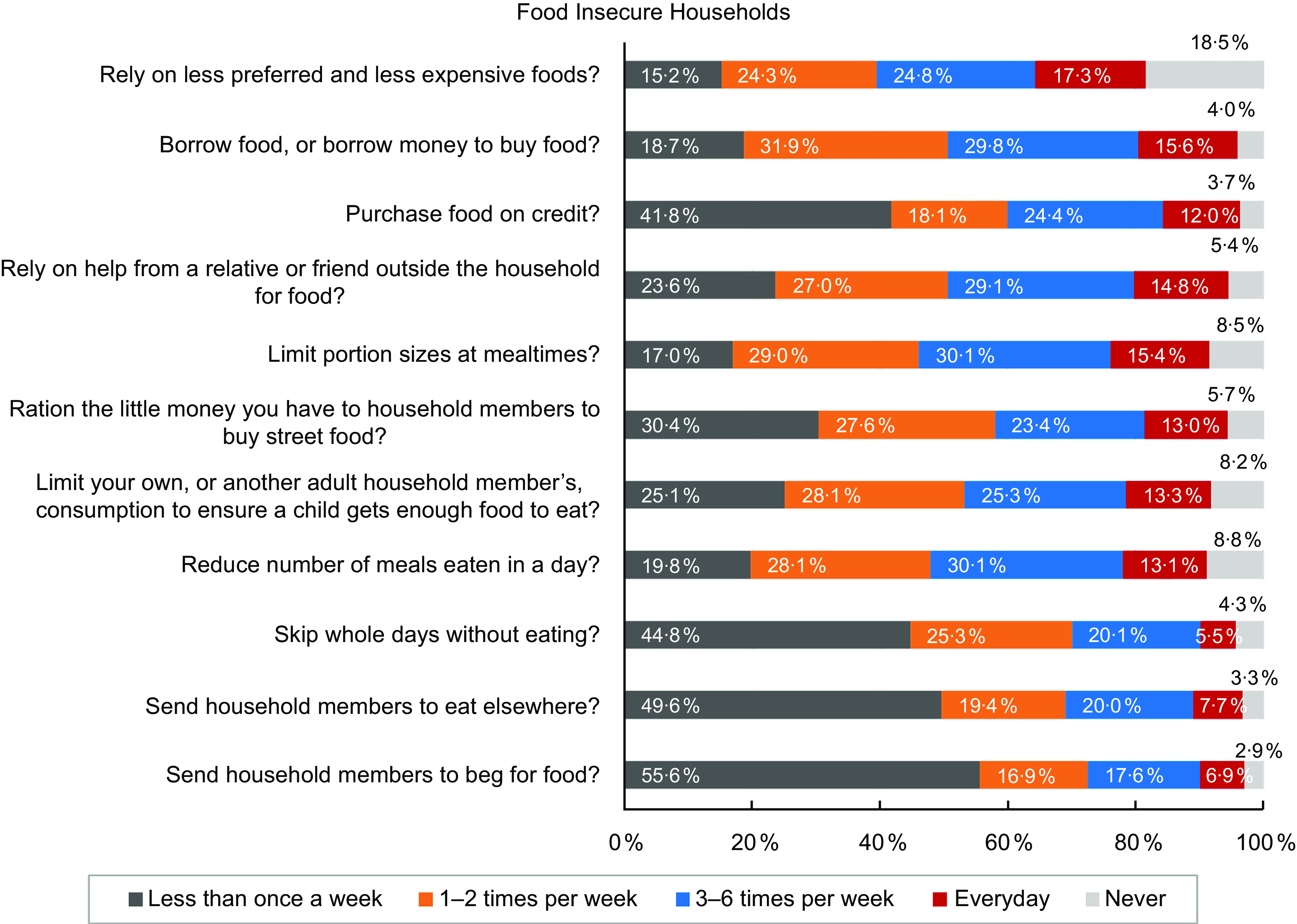
Coping strategies that prevailed among food-insecure households only

A comparison of the responses between food secure (Fig. 7) and insecure (Fig. 8) groups revealed that all these coping strategies were mostly used by the food insecure households.

### Prevalence of GAD-7 (anxiety) and PHQ-9 (depression) categories

The prevalence of minimal, mild, moderate and severe anxiety (GAD-7 categories) was about 59·1 %, 25·0 %, 12·7 % and 3·3 %, respectively, resulting in probable anxiety of 16·0 % (GAD-7 score > 10). In contrast, the prevalence of minimal, mild, moderate, moderately severe and severe depression (PHQ-9 categories) was approximately 49·4 %, 26·7 %, 15·9 %, 6·4 % and 1·6 %, respectively, resulting in 23·9 % of the respondents with probable depression (PHQ-9 score > 10).

### Associations of food insecurity and coping strategies with risk of anxiety and depression

**Fig. 9:**
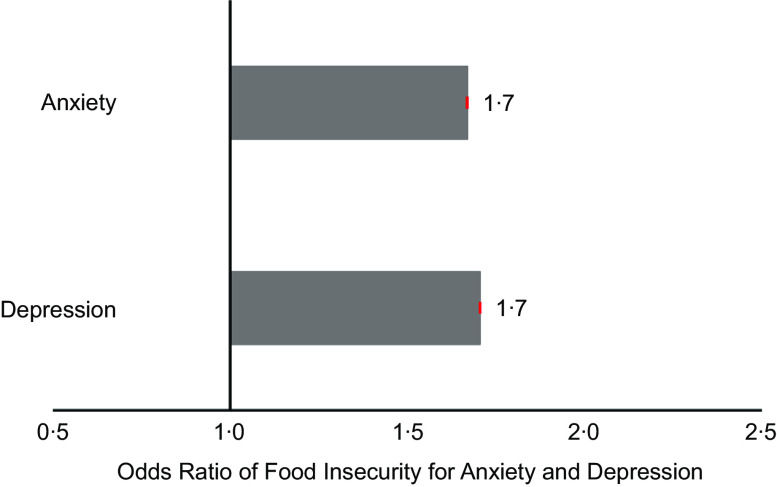
Associations of food insecurity with risk of anxiety and depression. Ordered logistic regression was used with food insecurity group as the predictor and Generalized Anxiety Disorder-7 (Anxiety) and Patient Health Questionnaire-9 (Depression) categories as the outcomes. Red error bars represent 95 % CI for the OR. The corresponding marginal effects as well as all values for the 95 % CI are presented in Table S1 of the supplementary data

The odds ratios of food insecurity for risk of anxiety and depression are shown in Fig. 9. The corresponding marginal effects as well as all values for the 95 % CI are presented in Table S1 of the supplementary data. Overall, one level increase in food insecurity (i.e. shifting from the ‘food secure’ to the ‘at risk’ or from the ‘at risk’ to the ‘food insecure’ group) was associated with 1·7 times greater odds of being in a higher anxiety or depression category (Fig. 9).

**Fig. 10:**
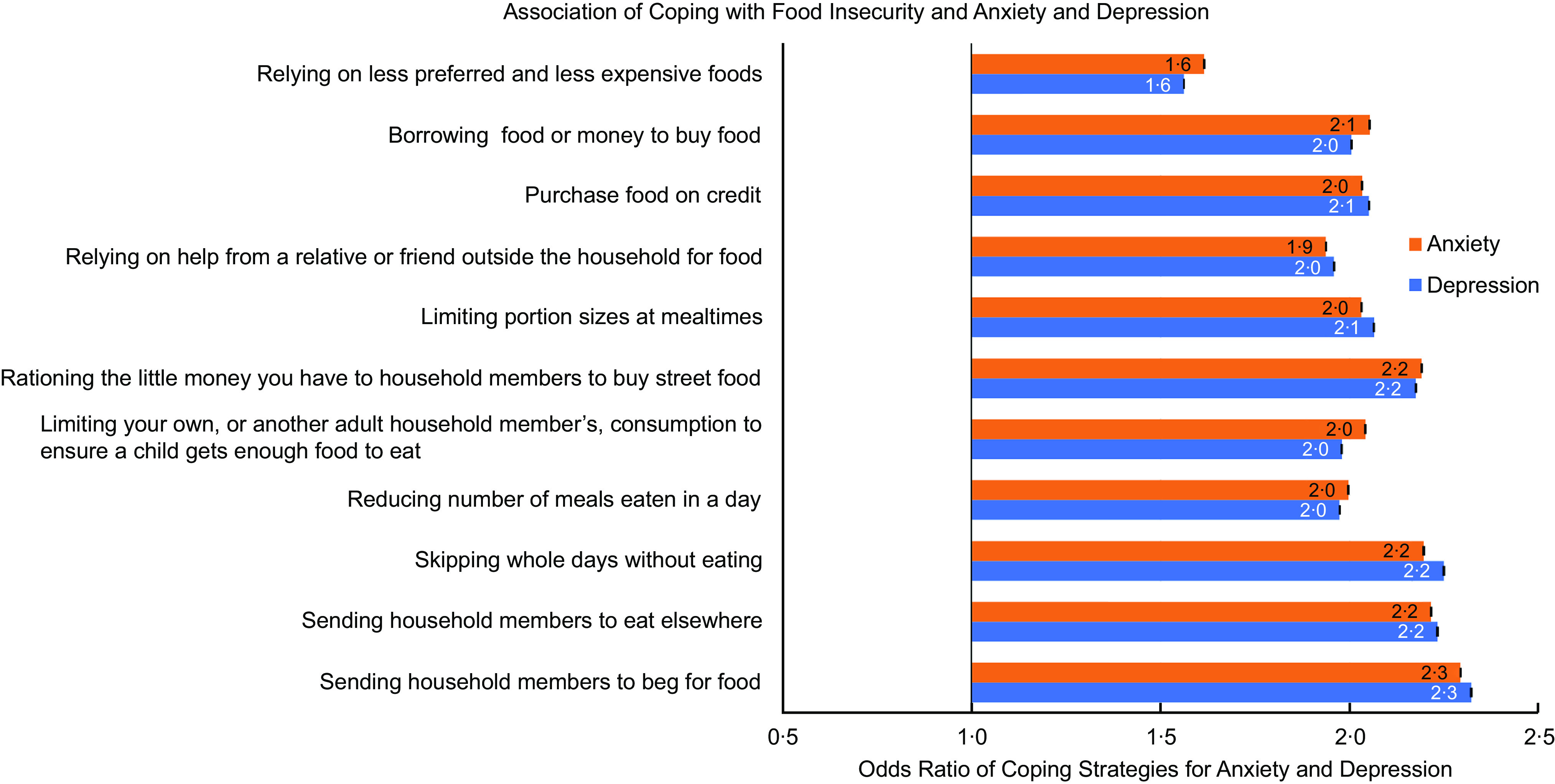
Associations between food insecurity coping strategies and anxiety and depression. Ordered logistic regression was used with each coping strategy as the predictor and Generalized Anxiety Disorder-7 (Anxiety) and Patient Health Questionnaire-9 (Depression) categories as the outcomes. Black error bars represent 95 % confidence intervals for the OR. The corresponding marginal effects as well as all values for the 95 % CI are presented in Tables S2 and S3 of the supplementary data

Figure 10 shows the OR of each coping strategy for the risk of anxiety and depression. The corresponding marginal effects as well as all values for the 95 % CI are shown in Tables S2 and S3 of the supplementary data. Overall, the odds for moving to a higher category of anxiety and depression differed among food insecurity coping strategies. However, the coping strategy associated with the highest odds (OR = 2·3) of moving to a higher category of anxiety and depression was ‘sending household members to beg for food’. Notably, after adjusting for the other coping strategies, the OR of anxiety and depression for sending household members to beg for food were 1·165 (95 % CIs = 1·164, 1·167) and 1·212 (95 % CIs = 1·211, 1·214), respectively (results not presented in Figures).

## Discussion

Recent studies from countries such as Bangladesh, Canada and the USA suggested that increased food insecurity during COVID-19 may be accompanied by an increased prevalence of impaired mental health^(17–19)^. In the present nationally representative survey, we investigated food insecurity and related coping strategies among South African households during COVID-19 (before the omicron wave 4 and while the country was in a low level of lockdown). We also tested the associations of food insecurity and related coping strategies with the risk of anxiety and depression. Although one in five South African households was food insecure, the prevalence varied widely across provinces, and households of low socio-economic status were the most affected. The most commonly used coping strategy was relying on less preferred and less expensive foods (46·0 %), while sending a household member to beg for food was the least used strategy (20·9 %). The use of multiple coping strategies was more common among food-insecure households compared to those who were food-secure, but many food secure households also employed strategies to stage off food insecurity. There was evidence to suggest that food insecurity and all its coping strategies were associated with greater odds of having anxiety and depression among South African household respondents.

The prevalence of food insecurity (20·4 %) was similar to that reported in 2017 (20·6 %)^(3)^ but lower than that from a recent national survey^(5)^ conducted during the height of the COVID-19 pandemic. In the recent NIDS-CRAM survey (wave 5), the prevalence of food insecurity was suggested to be at least 35·0 % among South African households^(5)^. However, there are some key discrepancies when comparing the survey designs between the NIDS-CRAM and the present study. In the NIDS-CRAM survey, food insecurity was defined as having run out of money to buy food in the previous month (March 2021). According to our definition, many of the respondents should have been classified as ‘at risk of food insecurity’ and two more questions would have been required to define food insecurity^(11)^. However, when using a definition similar to that used by NIDS-CRAM (proportion of households who had run out of money to buy food in the previous 30 d), the prevalence in our study was even lower (18·6 %). Furthermore, as the interviews were conducted telephonically in the NIDS-CRAM survey, households without telephones had been excluded^(5)^.

Our observation that the prevalence of food insecurity was dependent on the household’s province, community type and household asset score, as well as the respondent’s ethnicity, education level, employment status and monthly income, was in accordance with other nationally representative studies from both low- and high-income countries^(20–22)^. Likewise, these findings corroborate sub-national studies from South Africa where these socio-economic factors have been shown to associate with food insecurity^(23–25)^. Within the South African context, the high rates of food insecurity within poorer households have been primarily attributed to former apartheid regime policies that led to racial discrimination, geographic segregation and other unsustainable settlement patterns^(3,26)^.

While food insecurity coping strategies have been investigated by several sub-national South African surveys^(27–29)^, surveys that used nationally representative samples are lacking. Understanding food insecurity coping strategies at the national level is key when assessing the extent of food insecurity in South Africa, as these are strongly linked to socio-economic factors. For example, while relying on less preferred and less expensive foods is the most used strategy in South Africa, this coping strategy depends on the availability of those less preferred and cheaper foods^(30)^. Likewise, the ability to purchase food on credit may largely depend on the employment status^(31)^.

Furthermore, some of the coping mechanisms used by South African households may have significant malnutrition consequences for members of the households, for example, limiting portion sizes and skipping whole days without meals^(32,33)^. Similarly, begging for food may strongly exacerbate anxiety and depression, as suggested by the present study.

Independent of socio-economic factors, food insecurity has been consistently associated with poor mental health^(34–36)^. A global analysis of 149 countries demonstrated that food insecurity was associated with poorer mental health in a dose–response manner^(34)^, and a recent systematic review of African countries also demonstrated this association^(36)^. Although the relationship between food insecurity and the risk of poor mental health outcomes has been shown in some regions of South Africa^(37)^, evidence from nationally representative samples is limited. However, recent evidence from the NIDS-CRAM survey suggested that an increase in the prevalence of food insecurity during COVID-19 was accompanied by an increased risk of screening for depressive symptoms^(5)^. In the present nationally representative study, we have confirmed that food insecurity is associated with both anxiety and depression risk among South African adults. The causal association of food insecurity to mental health outcomes remains to be proven. However, it has been hypothesised that food insecurity may lead to anxiety and depression because of its association with some unfavourable experiences that lead to poor mental health^(34)^. Such experiences may include constant worrying about food, disruptions of meal patterns and acquiring food via socially unacceptable methods^(38)^. Notably, examples of these experiences form part of the list of coping strategies that were investigated in the present study. For example, disruptions of meal patterns may result from some of the tested coping strategies, including limiting portion sizes, reducing the number of meals eaten in a day and skipping whole days without eating. Similarly, begging for food and sending household members to eat elsewhere are considered by some as being socially unacceptable^(39,40)^.

Hence, our observation that the coping strategies were also associated with anxiety and depression, supports the hypothesis that the relationship between food insecurity and poor mental health may be partly mediated by coping strategies. Although mediation analysis was possible with our data, this was beyond the scope of the present study. Hence, further analyses are still needed to determine the mediation effects of each coping strategy. Importantly, findings from this study suggested that some of the coping strategies are more strongly associated with poor mental health than others. While begging for food was the least common coping strategy, it was identified as the strongest predictor of anxiety and depression risk. Conversely, while relying on less preferred and less expensive foods was the most prevalent coping strategy, it was less associated with the risk of anxiety and depression.

### Strengths and limitations

The key strength of the study was the use of a nationally representative sample and weighted data to represent the larger South African population. This study also has some limitations which should be considered when interpreting the findings. The study design was cross-sectional, and the causality of the observed relationships could not be inferred. Further research, including longitudinal studies and mediation analyses, is required to improve our understanding of the observed relationships.

## Conclusions

The prevalence of food insecurity in South Africa remains high, with at least one in five households affected. While the prevalence varies widely across provinces, households of low socio-economic status are the most affected. Collaborative efforts are needed from government and non-government agencies to assist those who are disproportionally affected. The coping strategies that are presently used by food-insecure households may have a serious negative impact on their mental health. Certainly, living in a food-insecure household is associated with a higher risk of anxiety and depression. This is the first study to show that the coping strategies used by food-insecure households are also associated with the risk of anxiety and depression.

### Recommendations

Our report comes timely with the recent announcement by the South African government to prioritise solutions that address the issues of unemployment, poverty and inequality. In the 2022 State of the Nation Address, the president stated that there would be several fundamental reforms primarily aimed at reviving economic growth in South Africa, which would ultimately assist in combating the challenges of high employment and food insecurity rates^(41)^. Within this context, the government aims to create conditions that will enable small and large businesses to emerge, grow, access new markets, create new products and hire more employees^(41)^.

Given the findings from the present study, we recommend that the South African government should also consider food insecurity and its associated coping strategies as risk factors for impaired mental health. Accordingly, the proposed public measures to reduce the prevalence of food insecurity may also improve the mental health quality of South Africans.

## Supplementary material

For supplementary material accompanying this paper visit https://doi.org/10.1017/S1368980023000186. click here to view supplementary material
