# Supplementary material for: Food insecurity and coping strategies and their association with anxiety and depression: a nationally representative South African survey
Source: Public Health Nutr. 2023 Jan 24;26(4):705–15. doi: 10.1017/S1368980023000186 (PMC10131152; doi:10.1017/S1368980023000186)
Supplement: Supplementary file 1 [file S1368980023000186sup.zip › S1368980023000186sup001.docx]

**Table S2 Association between food insecurity coping strategies and anxiety.**

| **Copying strategy** | **Odds ratio** | **Marginal effects for each anxiety (GAD-7) category** | | | |
| --- | --- | --- | --- | --- | --- |
|  |  | **Minimal** | **Mild** | **Moderate** | **Severe** |
| Relying on less preferred and less expensive foods | 1.615 (1.614 to 1.616) | 0.591 (0.591 to 0.591) | 0.249 (0.249 to 0.249) | 0.126 (0.126 to 0.127) | 0.033 (0.033 to 0.033) |
| Borrowing food or money to buy food | 2.053 (2.052 to 2.054) | 0.592 (0.592 to 0.592) | 0.249 (0.249 to 0.250) | 0.125 (0.125 to 0.126) | 0.033 (0.033 to 0.033) |
| Purchasing food on credit | 2.033 (2.032 to 2.034) | 0.592 (0.592 to 0.952) | 0.251 (0.250 to 0.251) | 0.125 (0.125 to 0.125) | 0.033 (0.033 to 0.033) |
| Relying on help from a relative or friend outside the household for food | 1.937 (1.936 to 1.938) | 0.591 (0.591 to 0.591) | 0.250 (0.250 to 0.250) | 0.126 (0.126 to 0.126) | 0.033 (0.033 to 0.033) |
| Limiting portion sizes at mealtimes | 2.031 (2.030 to 2.032) | 0.592 (0.592 to 0.593) | 0.250 (0.249 to 0.250) | 0.125 (0.125 to 0.125) | 0.033 (0.033 to 0.033) |
| Rationing the little money you have to household members to buy street food | 2.190 (2.189 to 2.192) | 0.592 (0.592 to 0.592) | 0.250 (0.250 to 0.250) | 0.124 (0.124 to 0.124) | 0.033 (0.033 to 0.033) |
| Limiting your own, or another adult household member’s, consumption to ensure a child gets enough food to eat | 2.042 (2.041 to 2.043) | 0.593 (0.592 to 0.593) | 0.250 (0.250 to 0.250) | 0.124 (0.124 to 0.124) | 0.033 (0.033 to 0.033) |
| Reducing number of meals eaten in a day | 1.997 (1.995 to 1.998) | 0.593 (0.592 to 0.593) | 0.249 (0.249 to 0.249) | 0.125 (0.125 to 0.125) | 0.033 (0.033 to 0.034) |
| Skipping whole days without eating | 2.197 (2.195 to 2.198) | 0.593 (0.593 to 0.593) | 0.251 (0.251 to 0.251) | 0.123 (0.123 to 0.123) | 0.033 (0.033 to 0.033) |
| Sending household members to eat elsewhere | 2.216 (2.214 to 2.217) | 0.594 (0.594 to 0.594) | 0.251 (0.250 to 0.251) | 0.123 (0.123 to 0.123) | 0.033 (0.033 to 0.033) |
| Sending household members to beg for food | 2.293 (2.292 to 2.295) | 0.594 (0.594 to 0.594) | 0.251 (0.251 to 0.251) | 0.122 (0.122 to 0.122) | 0.033 (0.033 to 0.033) |

Ordered logistic regression were used with each coping strategy as the predictor, and GAD-7 (Anxiety) categories as the outcomes. All P values were <0.001. The values in the parenthesis are 95% confidence intervals.
