# Supplementary material for: Food insecurity and coping strategies and their association with anxiety and depression: a nationally representative South African survey
Source: Public Health Nutr. 2023 Jan 24;26(4):705–15. doi: 10.1017/S1368980023000186 (PMC10131152; doi:10.1017/S1368980023000186)
Supplement: Supplementary file 1 [file S1368980023000186sup.zip › S1368980023000186sup002.docx]

**Table S1 Association between food insecurity and anxiety and depression.**

|  | **Odds ratio** | **Marginal effects for each category** | | | | |
| --- | --- | --- | --- | --- | --- | --- |
|  |  | **Minimal** | **Mild** | **Moderate** | **Moderately severe** | **Severe** |
| **Anxiety** | 1.669 (1.667 to 1.670) | 0.593 (0.593 to 0.593) | 0.249 (0.249 to 0.249) | 0.125 (0.125 to 0.126) | N/A | 0.033 (0.033 to 0.033) |
| **Depression** | 1.705 (1.704 to 1.706) | 0.497 (0.497 to 0.498) | 0.266 (0.266 to 0.266) | 0.157 (0.157 to 0.157) | 0.063 (0.063 to 0.064) | 0.016 (0.016 to 0.016) |

Ordered logistic regression were used with food insecurity group as the predictor, and GAD-7 (Anxiety) and PHQ-9 (Depression) categories as the outcomes. Values in the parenthesis are 95% confidence intervals. All P value were <0.001. **N/A:** Not Applicable.
