# Supplementary material for: Food insecurity and coping strategies and their association with anxiety and depression: a nationally representative South African survey
Source: Public Health Nutr. 2023 Jan 24;26(4):705–15. doi: 10.1017/S1368980023000186 (PMC10131152; doi:10.1017/S1368980023000186)
Supplement: Supplementary file 1 [file S1368980023000186sup.zip › S1368980023000186sup003.docx]

**Table S3 Association between food insecurity coping strategies and depression.**

| **Copying Strategy** | **Odds ratio** | **Marginal effects for each depression (PHQ-9) category** | | | | |
| --- | --- | --- | --- | --- | --- | --- |
|  |  | **Minimal** | **Mild** | **Moderate** | **Moderately severe** | **Severe** |
| Relying on less preferred and less expensive foods | 1.561 (1.561 to 1.562) | 0.494 (0.494 to 0.494) | 0.266 (0.266 to 0.266) | 0.159 (0.159 to 0.159) | 0.065 (0.065 to 0.065) | 0.167 (0.167 to 0.167) |
| Borrowing food or money to buy food | 2.005 (2.004 to 2.006) | 0.495 (0.495 to 0.495) | 0.266 (0.266 to 0.266) | 0.158 (0.158 to 0.158) | 0.064 (0.064 to 0.064) | 0.017 (0.017 to 0.017) |
| Purchasing food on credit | 2.051 (2.050 to 2.053) | 0.496 (0.495 to 0.496) | 0.268 (0.268 to 0.268) | 0.157 (0.157 to 0.158) | 0.063 (0.063 to 0.063) | 0.016 (0.016 to 0.016) |
| Relying on help from a relative or friend outside the household for food | 1.959 (1.957 to 1.960) | 0.496 (0.496 to 0.496) | 0.267 (0.267 to 0.267) | 0.157 (0.157 to 0.157) | 0.064 (0.064 to 0.064) | 0.017 (0.017 to 0.017) |
| Limiting portion sizes at mealtimes | 2.064 (2.063 to 2.066) | 0.495 (0.495 to 0.495) | 0.267 (0.267 to 0.267) | 0.157 (0.157 to 0.157) | 0.064 (0.064 to 0.064) | 0.017 (0.017 to 0.017) |
| Rationing the little money you have to household members to buy street food | 2.175 (2.174 to 2.177) | 0.497 (0.496 to 0.497) | 0.267 (0.267 to 0.267) | 0.156 (0.156 to 0.156) | 0.064 (0.064 to 0.064) | 0.017 (0.017 to 0.017) |
| Limiting your own, or another adult household member’s, consumption to ensure a child gets enough food to eat | 1.979 (1.978 to 1.980) | 0.497 (0.497 to 0.497) | 0.267 (0.267 to 0.268) | 0.156 (0.155 to 0.156) | 0.064 (0.063 to 0.064) | 0.017 (0.017 to 0.017) |
| Reducing number of meals eaten in a day | 1.973 (1.972 to 1.975) | 0.496 (0.496 to 0.496) | 0.267 (0.267 to 0.267) | 0.156 (0.156 to 0.126) | 0.064 (0.064 to 0.064) | 0.017 (0.017 to 0.017) |
| Skipping whole days without eating | 2.249 (2.247 to 2.250) | 0.495 (0.495 to 0.495) | 0.268 (0.268 to 0.268) | 0.156 (0.156 to 0.157) | 0.064 (0.064 to 0.064) | 0.017 (0.017 to 0.017) |
| Sending household members to eat elsewhere | 2.233 (2.231 to 2.235) | 0.496 (0.495 to 0.496) | 0.268 (0.268 to 0.268) | 0.156 (0.156 to 0.156) | 0.063 (0.063 to 0.063) | 0.017 (0.017 to 0.017) |
| Sending household members to beg for food | 2.322 (2.320 to 2.324) | 0.497 (0.497 to 0.497) | 0.269 (0.269 to 0.269) | 0.155 (0.155 to 0.155) | 0.063 (0.062 to 0.063) | 0.017 (0.017 to 0.017) |

Ordered logistic regression were used with each coping strategy as the predictor, and PHQ-9 (Depression) categories as the outcomes. All P values were <0.001. The values in the parenthesis are 95% confidence intervals.
